# Usefulness of advanced monoenergetic reconstruction technique in dual-energy computed tomography for detecting bladder cancer

**DOI:** 10.1007/s11604-021-01195-5

**Published:** 2021-09-13

**Authors:** Motoo Nakagawa, Taku Naiki, Aya Naiki-Ito, Yoshiyuki Ozawa, Masashi Shimohira, Masahiro Ohnishi, Yuta Shibamoto

**Affiliations:** 1 Department of Radiology, Nagoya City University Graduate School of Medical Sciences, 1 Kawasumi, Mizuho-cho, Mizuho-ku, Nagoya, Japan; 2 Department of Nephro-Urology, Nagoya City University Graduate School of Medical Sciences, Nagoya, Japan; 3 Department of Experimental Pathology and Tumor Biology, Nagoya City University Graduate School of Medical Sciences, Nagoya, Japan

**Keywords:** Bladder cancer, Dual energy CT, Advanced virtual monoenergetic image

## Abstract

**Purpose:**

Detecting bladder cancer (BC) in routine CT images is important but is sometimes difficult when cancer is small. We evaluated the ability of 40-keV advanced monoenergetic images to depict BC.

**Materials and methods:**

Fifty-two patients with a median age of 74 years (range 45–92) who were diagnosed as BC with transurethral resection or cystectomy, were included. They were examined with contrast-enhanced dual-energy CT (DE-CT) and advanced virtual monoenergetic images (40 keV) were reconstructed. For evaluating depictability of BC on 40-keV or virtual-120-kVp images, the difference in CT number between the cancer and bladder wall (BC–BW value) were calculated. We also subjectively assessed depictability of BC in virtual-120-kVp and 40-keV images using a 4-grade Likert scale (3: clear, 0: not visualized).

**Results:**

In 42 of 52 patients, BC–BW values could be calculated because BC was detected on CT images. The mean BC–BW value at 40 keV was significantly higher than that of virtual 120 kVp [80.5 ± 54 (SD) vs. 11.4 ± 12.5 HU, *P* < 0.01]. Average scores of subjective evaluations in the virtual-120-kVp and 40-keV images were 1.7 ± 1.2 and 2.1 ± 1.2, respectively (*P* < 0.001).

**Conclusion:**

The advanced monoenergetic reconstruction technique reconstructed using DE-CT image is useful to depict BC.

## Introduction

Every year, 27 million bladder cancer (BC) patients are diagnosed worldwide [1]. Gross hematuria is the most common symptom of BC and patients with gross hematuria are four times more likely to have malignancy than those with microhematuria. Some BC patients do not have symptoms such as hematuria. Bladder cancer in those patients could be found incidentally on routine abdominal contrast-enhanced computed tomography (CE-CT).

Contrast-enhanced CT is a useful modality for detecting BC [2, 3]. Currently, CT urography replaces intravenous urography as the first choice for the evaluation of urinary tract tumors [4–6]. CT urography is defined as CT examination of the kidneys, ureters and bladder with at least one series of images acquired during the excretory phase after intravenous contrast administration [7].

However, small BCs are sometimes hard to be depicted by conventional routine CE-CT scanned at 120 peak kilo electron volt (kVp) [during the nephrogenic phase (80–100 s)] due to lower enhancement. Although excretory phase images are also useful to detect BC [8], the detection rate of bladder tumor in the excretory phase is not very high, due to the fluid level in this phase causing some anterior wall bladder lesions to be missed [3, 8, 9].

There have been some trials to detect bladder tumors in the portal phase image (60–80 s delay scan), with reports of detection rate higher than that of excretory phase [10, 11]. On the other hand, only a few reports are available for the nephrographic phase image (80–100 s), since small BCs are sometimes hard to be depicted due to lower enhancement [12]. However, the nephrogenic phase image is generally used as routine CE-CT images, practically.

There have been several types of dual energy CT (DE-CT) (e.g., photon-counting, fast kVp-switching, multilayer detectors, etc.). DE-CT of SOMATOM Definition Flash (Siemens Healthineers, Forchheim, Germany) has two X-ray tubes with different energies. Monoenergetic images can be reconstructed using two different energies. Contrast of lower kilo electron volt (keV) images is stronger than that of usual low-kVp images such as 80-kVp images [13]. Therefore, we considered sensitivity of BC detection could be increased if low-keV image is used as routine CE-CT examination (100 s). In this study, we evaluated the detectability of 40-keV monoenergetic images (100 s) for BC.

## Methods and materials

This retrospective study evaluated the diagnostic value of monoenergetic imaging for BC and clinical information at our medical center, and was approved by the Institutional Review Board, which waived the requirement for informed consent.

### Patients

Fifty-two patients who were diagnosed as BC with transurethral resection (TUR; *n* = 42, 81%) or cystectomy in (*n* = 10, 19%) and underwent DE-CT were included.

There were 40 (77%) men and 12 (23%) women, and their median age was 74 years (range 45–92).

### Histopathologic analysis

The specimens were examined with hematoxylin–eosin staining for histopathologic evaluation. All BCs were staged by a pathologist with an experience of 14 years in accordance with American Joint Committee on Cancer (AJCC) Staging Manual, 8th edition published in 2017 [14] and were defined using World Health Organization classification published in 2016 [15].

### DE-CT

Somatom Definition Flash was used for all patients with 100-kVp and 140-kVp tube voltages and a 0.9 pitch. Acquisition collimation was set to 32 × 0.6 mm. Advanced virtual monoenergetic images (40 keV) and virtual images corresponding to a 120-kVp scan were reconstructed for each patient. The 40-keV image is the lowest energy image which can be reconstructed with DE-CT of Somatom Definition Flash. All imaging data were reconstructed with a slice thickness of 1.0 mm at a 0.8-mm increment. Dual energy scans were used for nephrogenic phases and usual single energy scan (120 kVp) were used for excretory phases.

### Nephrogenic phase and excretory phase images

A contrast medium (300 mgI/mL, 100 mL) was injected through an upper extremity vein at a flow rate of 2.0 mL/s. Scanning was performed 100 s and 15 min after injection of contrast material is started as a nephrogenic phase and excretory phase, respectively. To clearly depict BC by expanding the bladder at the excretory phase, patients were given 500 mL of fresh water before CT examination. The scan range of DE-CT included from the upper margin of the liver to the lower margin of the ischium. The excretory phases were scanned from the upper renal margin to the lower urethral margin.

### Subjective image evaluation

We evaluated how clearly the BCs were depicted in virtual-120-kVp, 40-keV, and excretory phase 120-kVp images.

Grade 3: clear.

Grade 2: mildly blurred but adequately diagnostic.

Grade 1: nondiagnostic due to blurring tumors.

Grade 0: not visualized.

Grade 3 and 2 were considered as “depicted”. Two board-certified radiologists were blinded to the imaging techniques and evaluated the images individually. The locations of BCs were not told to two radiologists before image evaluation. During image evaluation, window level (WL) and width (WW) were fixed for virtual 120 kVp (WL 30, WW 300) and for 40 keV (WL 100, WW 700). If WW is narrowed, tumor depictability probably can be increased. However, the purpose of this study was to evaluate the usefulness of reconstructed low-keV images as routine CT examination for detecting BC. Images with too narrow WW are not suitable for routine CT examination due to invisibility of other organs and vessels. Therefore, WW and WL of virtual-120-kVp and 40-keV images were adapted to be useful for routine imaging use which can distinguish air from fat, the hepatic vein from hepatic parenchyma, etc. Multiplanar reconstruction also could be used freely. For excretory phase, WW and WL could be changed freely when images were evaluated. Any discrepancies were resolved by consensus.

### Objective image evaluation

Region of interests (ROI) were set on the center of BC and bladder wall (BW) on CE-CT images of virtual 120 kVp and 40 keV. The CT numbers and image noise (standard deviation SD) were calculated as the average of four independent measurements by two readers. Then, we calculated the difference between the CT numbers of the BC and BW (BC–BW value). BC–BW values calculated from virtual-120-kVp and 40-keV images were compared. An imaging protocol which had a larger BC–BW value was considered a better modality for BC. When the bladder had multiple lesions, the largest cancer was evaluated with subjective and objective image evaluation. The average sizes of the ROI of BC and BW were also recorded.

Image noises of 40-keV and virtual-120-kVp images were evaluated with the SD of the CT numbers of BC and BW. Then, the SDs of 40-keV and virtual-120-kVp images were compared. The contrast-to-noise ratio (CNR) was calculated as (average attenuation of BC–average attenuation of BW) divided by the image noise of BW.

### Radiation dose

We recorded the computed tomography dose index (CTDI) and the dose-length product (DLP) of the DE-CT and excretory-phase-120kVp scan with the values displayed on the CT system (phantom size is 32 cm). The effective doses (ED) for the DE-CT and excretory-phase-120 kVp scan were calculated using the following equation: ED = *k* × DLP. *K* is 0.015 mSv mGy^−1^ cm^−1^, which is a conversion coefficient for the adult abdomen [16].

### Statistical analysis

The Shapiro–Wilk *W* test was used to determine whether CT numbers of the BC, urine, and bladder wall were normally distributed. Parametric data were compared using the paired *t* test. The non-parametric subjective data of image findings of BC were compared using the Wilcoxon signed rank test. For this subjective evaluation, interobserver agreement was calculated using kappa statistics. Kappa scores of 0.41–0.60, 0.61–0.80, and > 0.80 were considered to indicate moderate, good, and excellent agreement, respectively.

## Results

### Tumor characteristics

Tumor stages were histologically confirmed in 52. The case numbers of pathological T (pT) stages are listed in Table 1. The tumors measured 0.4–32.0 mm in maximum diameter (mean, 10.5 mm). Histologic diagnoses were non-invasive papillary urothelial carcinoma, low-grade (*n* = 23), non-invasive papillary urothelial carcinoma, high-grade (*n* = 5), urothelial carcinoma in situ (*n* = 3), invasive urothelial carcinoma (*n* = 17), and invasive urothelial carcinoma with divergent differentiation (*n* = 4).

**Table 1:** Pathological T (pT) stages

pT stage	*n*
Tis	5
Ta	26
T1	12
T2a	2
T2b	1
T3a	3
T3b	2
T4a	1
T4b	0
Total	52

### Subjective image evaluation

**Fig. 1:**
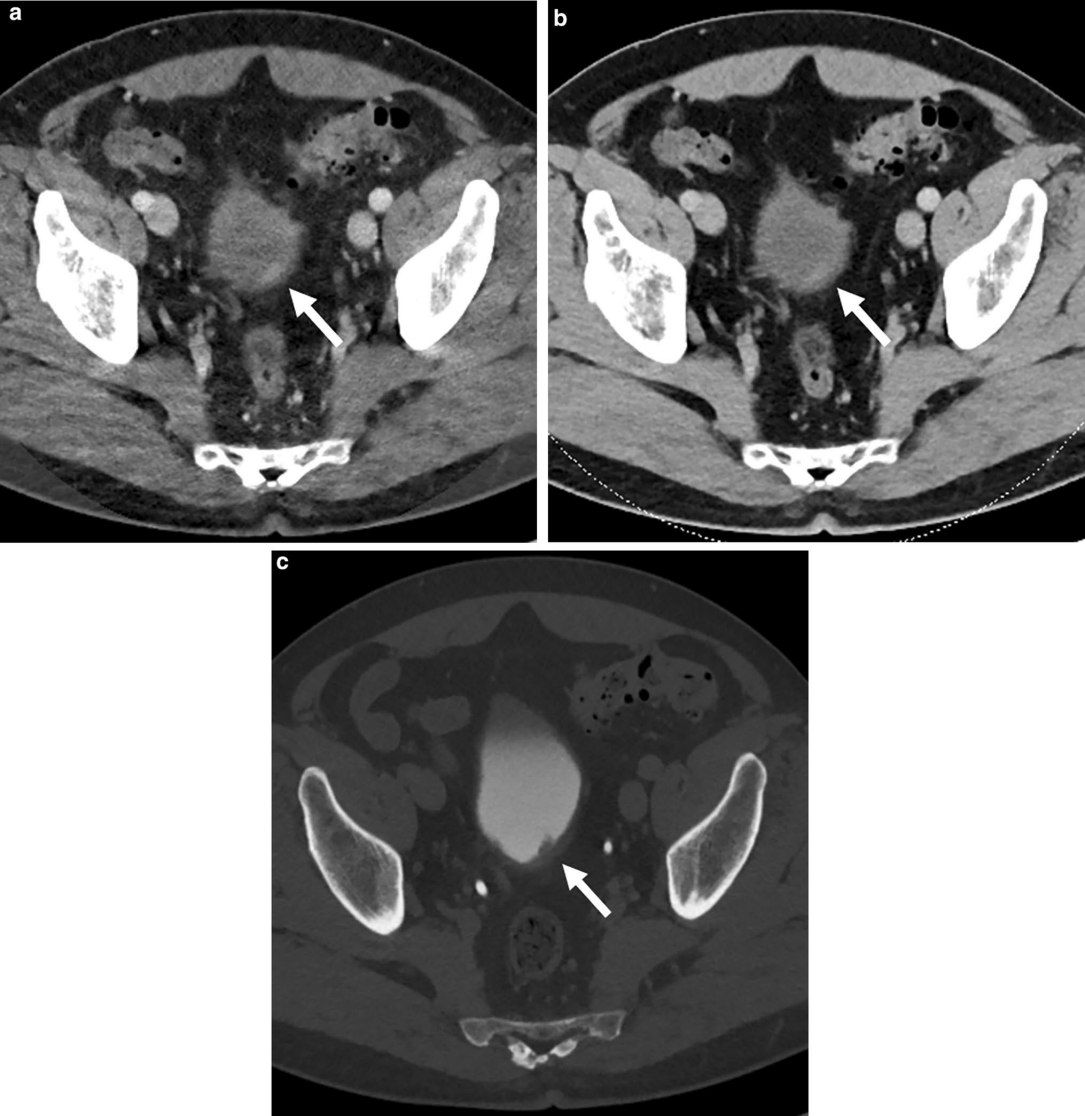
Stage pT1 invasive urothelial carcinoma in a 61-year-old man. Transverse CT images (**a** 40 keV, **b** 120 kVp, **c** excretory phase 120 kVp) show a mass (arrows) on the posterior bladder wall. The mass is more clearly depicted on 40-keV than on 120-kVp images (40 keV, grade 2; 120 kVp, grade 1; excretory phase, grade 3)

Of the 52 patients, BCs were depicted (grade 3 or 2) in 28 (53%), 40 (77%), and 32 (62%) cases on virtual-120-kVp, 40-keV, and excretory-phase 120-kVp images, respectively (Fig. 1). The grades of image quality for the BCs are shown in Table 2. Average scores of the virtual-120-kVp, 40-keV, and excretory-phase images were 1.7 ± 1.2 (SD), 2.1 ± 1.2, and 1.8 ± 1.3, respectively (Table 2). There was a significant difference between grades of 40-keV and virtual-120-kVp images (*P* < 0.001). Between the excretory-phase and both of the 40-keV and virtual-120-kVp images, there were no significant differences (*P* = 0.098 and 0.18). One case had contrast-material accumulation in the bladder at nephrogenic phase. However, it did not interfere with the diagnosis because tumor of the case was not small (15 mm). Inter-observer agreement was excellent (Kappa = 0.80, 0.85, and 0.91 for virtual-120-kVp, 40-keV, and excretory-phase images, respectively).

**Table 2:** Image quality of virtual-120-kVp, 40-keV, and excretory-phase images for bladder cancer

	120 kVp	40 keV	Excretory phase	40 + E	120 + E
Grade 3	21	27	26	32	29
Grade 2	7	13	6	10	6
Grade 1	13	2	6	1	7
Grade 0	11	10	14	9	10
Average grade (± SD)	1.7 ± 1.2	2.1 ± 1.2	1.8 ± 1.3	2.3 ± 1.1	2.0 ± 1.2

40 + E: combination of 40-keV and excretory-phase images

120 + E: combination of virtual-120-kVp and excretory-phase images

*SD* standard deviation

**Fig. 2:**
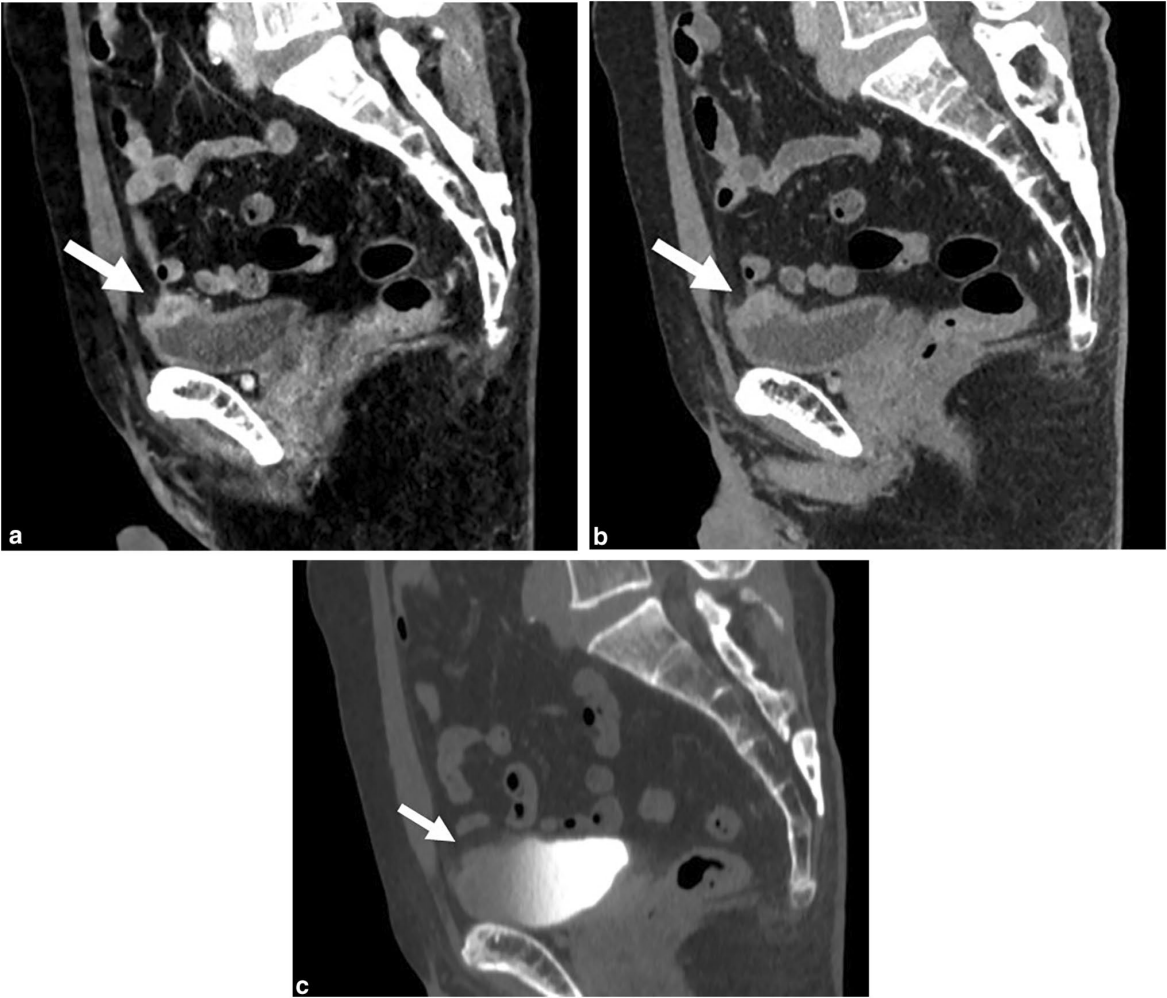
Stage pT1 invasive urothelial carcinoma with divergent differentiation in a 66-year-old man. Sagittal CT images (**a** 40 keV, **b** 120 kVp, **c** excretory phase 120 kVp) show a mass (arrows) on the dome of the bladder. The mass is not depicted on the excretory phase image because the anterior part of the inside of the bladder is not enhanced. The mass is more clearly depicted on 40-keV than on 120-kVp images (40 keV, grade 3; 120 kVp, grade 2; excretory phase, grade 0)

The score evaluated using images of both 40 keV and excretory phase (40 + E) was 2.3 ± 1.1. The score of both virtual-120 kVp and excretory phase (120 + E) was 2.0 ± 1.2. There was a significant difference between the score of 40-keV images alone and that of 40 + E images (*P* = 0.039). There was also a significant difference between the results of 40 + E and 120 + E (*P* = 0.002). Two cases (3.8%) were not depicted with the virtual-120-kVp or 40-keV images but depicted with the excretory-phase images. Ten cases (19%) were not depicted with excretory-phase images but depicted with 40-keV images (Fig. 2).

### Objective image evaluation

Forty-two BCs which were graded as 1–3 in subjective 40-keV image evaluation were evaluated with CT numbers, SD and ROI. These results are summarized in Table 3. There were five cases of multiple BC. The largest masses selected for objective image evaluation were consistent in all five cases. The mean CT number of the BC at 40 keV was significantly higher than that of virtual 120 kVp (167 ± 54 vs. 71.5 ± 163 HU, *P* < 0.01). The mean CT number of the BW at 40 keV was significantly higher than that of virtual 120 kVp (86.4 ± 24 vs. 60.1 ± 9.4 HU, *P* < 0.01). The mean BC–BW value at 40 keV was significantly higher than that of 120 kVp (80.5 ± 54 vs. 11.4 ± 12.5 HU, *P* < 0.01). The SDs of BC and BW of virtual-120-kVp images were significantly lower than that of 40-keV images (Table 3). The contrast-to-noise ratio of virtual-120-kVp and 40-keV image was 1.38 and 6.77, respectively.

**Table 3:** Objective evaluation of virtual-120-kVp and 40-keV images

	Virtual 120 kVp		40 keV	
	Bladder cancer	Bladder wall	Bladder cancer	Bladder wall
CT number (H.U.)	71.5 ± 163	60.1 ± 9.4	167 ± 54	86.4 ± 24
SD	8.0 ± 2.5	6.8 ± 4.9	26.8 ± 8.2	17.7 ± 8.1
ROI (mm^2^)	17.3 ± 13.2	5.7 ± 4.2	17.3 ± 13.2	5.7 ± 4.2
CNR	1.38		6.77	

*CT* computed tomography, *H.U.* Hounsfield unit, *SD* standard deviation, *ROI* region of interest, *CNR* contrast-to-noise ratio

### Radiation dose

Computed tomography dose index and DLP of DE-CT were 9.6 ± 0.67 mGy and 545 ± 131 mGy cm, respectively. Those of excretory-phase-120-kVp scan were 13.4 ± 4.0 mGy and 500 ± 160 mGy cm (*P* > 0.05), respectively. Effective doses of the DE-CT and excretory-phase-120kVp scan were 8.2 ± 2.0 mSv and 7.5 ± 2.4 mSv, respectively (*P* > 0.05).

## Discussion

Malignant bladder lesions are incidentally detected on CE-CT images [17]. Although CT is a useful imaging modality, BC is sometimes hard to be detected with routine CT examination for screening, searching metastasis, etc. due to reasons such as small cancer size and low attenuation. Therefore, excretory-phase images are needed to more efficiently detect BC [3]. However, excretory phase imaging is usually omitted unless problems of urinary tract are suspected. Even if the excretory phase is scanned, small BCs at the anterior wall sometimes cannot be detected. The anterior wall of the bladder is sometimes not enhanced because CT contrast materials tend to be deposited. Although MRI and bladder cystoscopy are useful modalities for diagnosis of BC, long examination time and invasiveness are problematic, respectively. Therefore, a scan protocol that has a high sensitivity for BC is desirable for routine CE-CT.

CT images are generally obtained with a tube voltage of 120 kVp in adults. The X-rays generated by these tube voltages are polyenergetic, and the X-ray spectrum contains the continuous spectrum and the line spectrum. The “p” of kVp is the “peak”, and the number before the unit represents the maximum energy. In other words, the maximum energy of 120 kVp X-rays is 120 kV, but other lower energy components are also included. With dual energy scan technique, a virtual monoenergetic imaging can be reconstructed as arbitrary monoenegy (40, 50, 70, and 100 keV, etc.). It is also called a virtual monochromatic image [18], which is similar to extracting monochromatic light from visible white light. The unit of voltage in these virtual monochromatic energy images is represented by “keV “, which seems to suggest monochromatic energy that is not a continuous energy spectrum.

In low-keV images that are reconstructed using the virtual monoenergetic technique, contrast enhancement can be strengthened compared with usual CE-CT images, as shown in our and previous studies [13]. Using monoenergetic-image reconstruction, the BC–BW value of the 40-keV image was higher than that of the virtual-120-kVp image. Because contrast between BC and BW was strengthened by 40-keV-image reconstruction, BC depictability and CNR of 40-keV images were higher than those of virtual-120-kVp images study even though image noise (SD) of 40-keV images were higher than that of virtual-120-kVp images. The reason for these results is that the 40 keV image has a higher contrast effect (Table 3). In our study, BC detectability of 40 + E was also better than that of 120 + E. Because there were many patients with lower pT stage (pT < 1: 81%) in our study, the 40-keV image is considered a sensitive modality for detecting BC especially in early stage. Therefore, monoenergetic imaging can be useful for routine CT examination in detecting small BC even in asymptomatic patients.

Depictability of 40 + E was higher than that of the 40-keV image alone. Even if DE-CT is scanned and 40-keV images are reconstructed, excretory-phase images may be preferred to be obtained for diagnosing BC. However, including the excretory phase in routine imaging may be somewhat difficult in daily clinical practice due to a longer examination time.

There were a few limitations in this study. Normal cases and patients with inflammatory bladder disease or benign bladder tumor were not included. Therefore, false positive rate and specificity of monoenergetic imaging was not evaluated. In this study, we compared 40-keV and virtual-120kVp images, not true-conventional-120kVp images. Therefore, the results might be slightly different from this study if compared to actual-120 kVp images with 40-keV images. Some studies revealed that radiation doses of DE-CT examination for monoenergetic imaging were same as those of usual examination using CT of one generation ago [19, 20]. However, radiation doses of DE-CT can be higher than those of low-kVp scans (e.g. 70 or 80 kVp) with a latest CT scanner.

## Conclusion

BC detectability of 40-keV images reconstructed using DE-CT is higher than that of virtual-120-kVp images. Low-keV monoenergetic imaging may have a potential as practical routine CT imaging for the detection of bladder cancer.
